# Chondroid syringoma of the upper lip: a rare case highlighting diagnostic challenges and literature review

**DOI:** 10.1093/jscr/rjaf541

**Published:** 2025-07-17

**Authors:** Amine Oussalem, Bouchra Dani, Malik Boulaadas

**Affiliations:** Specialty Hospital, Department of Maxillo-Facial Surgery, Area Lamfadel Cherkaoui, Rabat - Institut B.P 6527, Rabat, Morocco; Specialty Hospital, Department of Maxillo-Facial Surgery, Area Lamfadel Cherkaoui, Rabat - Institut B.P 6527, Rabat, Morocco; Specialty Hospital, Department of Maxillo-Facial Surgery, Area Lamfadel Cherkaoui, Rabat - Institut B.P 6527, Rabat, Morocco

**Keywords:** chondroid syringoma, cutaneous adnexal tumor, upper lip, immunohistochemistry, surgical excision, case report

## Abstract

Chondroid syringoma is a rare benign cutaneous adnexal tumor, most commonly found in the head and neck region. Its occurrence on the upper lip is exceptional. We report a case involving a 63-year-old man presenting with a painless, firm swelling of the left upper lip that gradually enlarged over three years. Clinical examination revealed a 1.6 cm submucosal nodule. Surgical excision with a 3 mm margin and direct closure using 5–0 Vicryl Rapide sutures was performed. Histopathological and immunohistochemical analysis confirmed the diagnosis of chondroid syringoma. No recurrence or functional/aesthetic complications were observed during 12 months of follow-up. This report underscores the diagnostic difficulties posed by this rare location, discusses differential diagnoses with other adnexal and salivary gland tumors, and reviews management considerations based on current literature.

## Introduction

Chondroid syringoma (CS) is a rare benign mixed tumor of sweat gland origin, characterized by epithelial and mesenchymal components. First described in 1961, it accounts for <0.01% of cutaneous tumors [1]. CS primarily affects the head and neck region, particularly the scalp, cheek, and nose [2]. However, localization to the upper lip is exceedingly rare, with only a handful of cases reported in the literature.

Due to its slow growth and non-specific clinical appearance, CS often mimics other benign cutaneous or salivary gland tumors, which complicates preoperative diagnosis. We report a rare case of a chondroid syringoma located in the upper lip of a 63-year-old male, emphasizing the clinical presentation, histopathological features, and surgical management, and we discuss its relevance in the context of the existing literature.

## Case report

**Figure 1:**
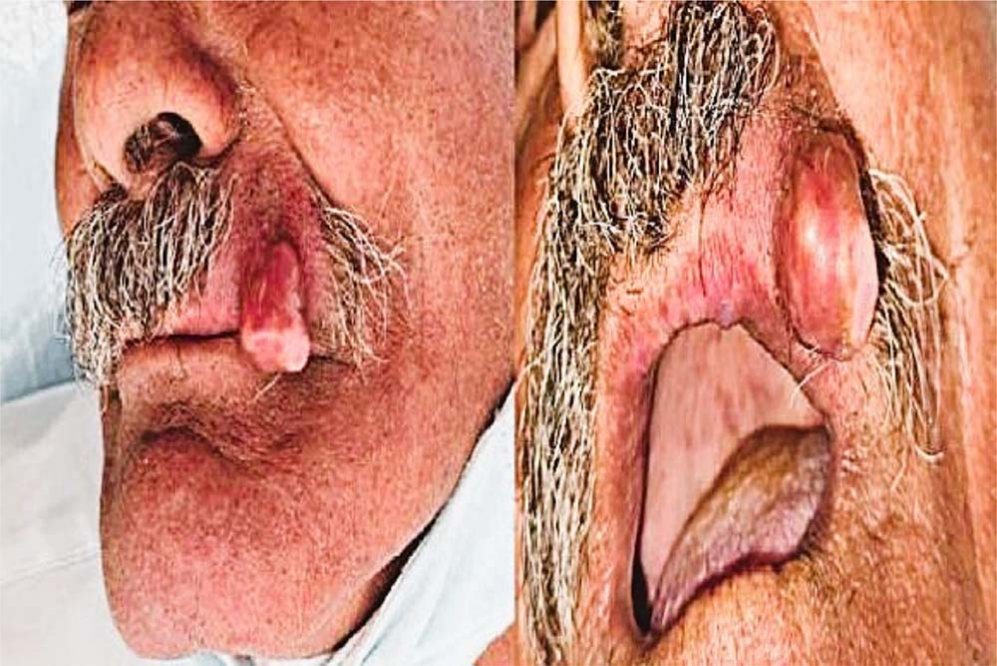
Preoperative clinical image showing a firm, non-tender nodule on the left upper lip.

A 63-year-old man with no significant medical history presented with a painless, slowly enlarging swelling on the left upper lip, first noticed three years prior. The mass had grown progressively without discomfort, bleeding, or ulceration. Examination showed a firm, mobile 1.6 cm submucosal nodule with intact overlying tissues. No cervical lymphadenopathy was present (Fig. 1).

**Figure 2:**
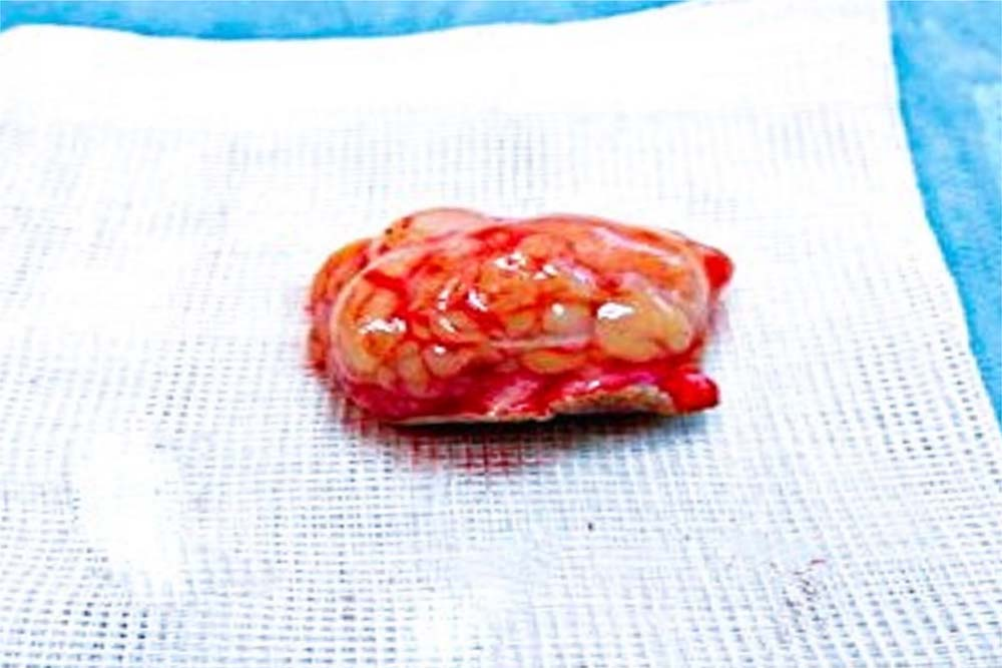
Macroscopic image showing the tumor.

Due to its small size and superficial location, no preoperative imaging was performed. Under local anesthesia, complete en bloc excision with 3 mm margins was performed and the wound was closed with interrupted 5–0 Vicryl Rapide sutures selected for its rapid absorption (50–80% tensile strength loss by day 10) and reduced risk of tissue reactivity in aesthetic zones [3]. Macroscopically, the mass was a firm, encapsulated nodule measuring 1.8 × 1.5 × 1.3 cm (Fig. 2).

Histopathological analysis revealed a well-circumscribed dermal tumor composed of epithelial and myoepithelial cells arranged in nests and tubules embedded in a chondromyxoid stroma. The epithelial cells displayed bland cytology without atypia or mitotic figures. No infiltrative growth was observed.

**Figure 3:**
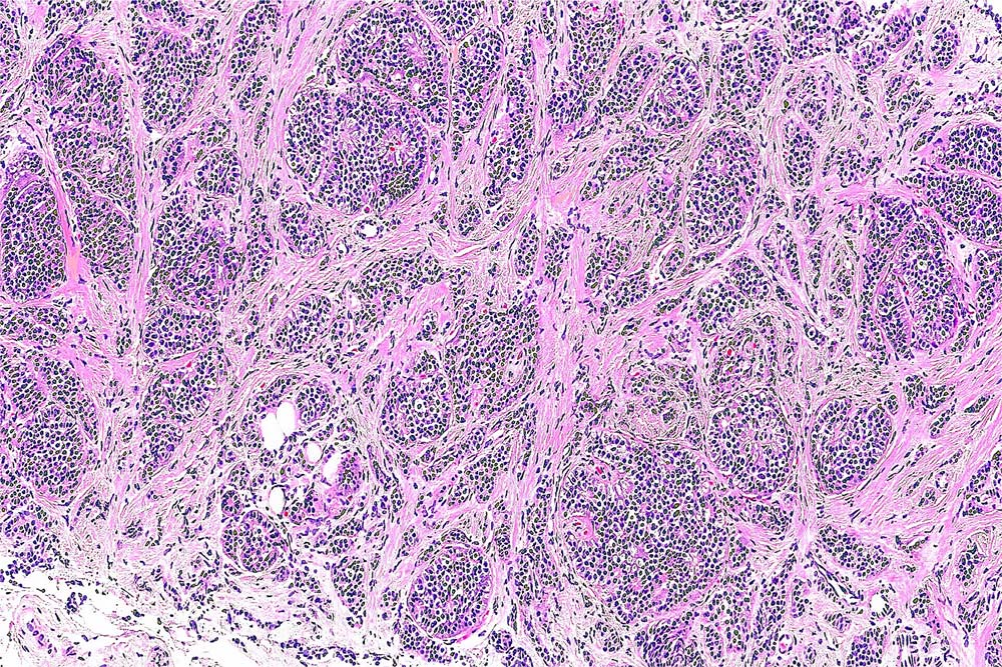
Histological section showing a well-circumscribed tumor composed of epithelial nests and tubules embedded in a myxoid and chondroid stroma (H&E, original magnification ×100).

Immunohistochemistry confirmed the diagnosis, with strong S-100 protein expression in myoepithelial cells and cytokeratin AE1/AE3 positivity in epithelial components (Fig. 3).

**Figure 4:**
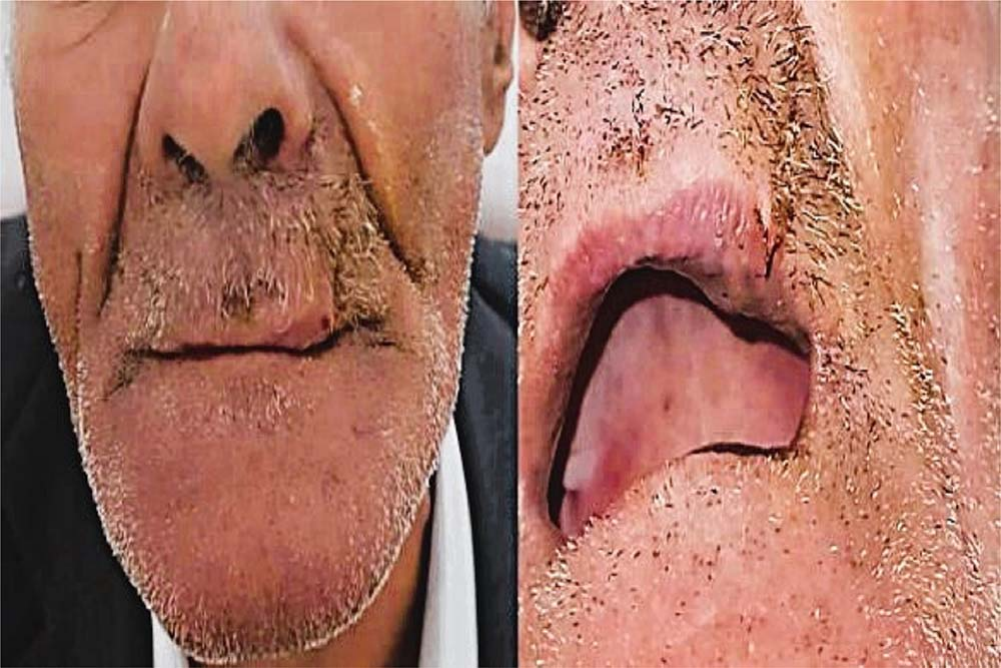
12-month postoperative result showing no recurrence and optimal aesthetic outcome.

Surgical margins were clear. The postoperative period was uneventful, with good wound healing, preserved upper lip mobility, and a favorable aesthetic outcome. Follow-up at 12 months revealed no signs of recurrence or complications (Fig. 4).

## Discussion

Chondroid syringoma is a rare benign adnexal neoplasm that most commonly arises in the head and neck, but involvement of the upper lip is exceptional [1, 2]. Its rarity at this location contributes to the diagnostic challenge, as it may be mistaken for salivary gland tumors such as pleomorphic adenoma or other adnexal tumors like cylindroma and spiradenoma [4].

Table 1 summarizes reported upper lip CS cases since 2000 [2, 5–7]. Our case shows typical features: size <2 cm, male predominance (73%), and narrow-margin excision (2–5 mm). Recurrence is exceptional without incomplete excision.

**Table 1:** Reported cases of chondroid syringoma of the upper lip

**Author/Year**	**Age/Sex**	**Size**	**Margins**	**Outcome**
Goyal 2020 [2]	55/M	1.2 cm	2 mm	NED, 18 months
Lee 2017 [5]	48/F	1.5 cm	3 mm	NED, 24 months
Rossi 2015 [6]	60/M	2.0 cm	5 mm	Recurrence, 12 months
**Our case**	**63/M**	**1.6 cm**	**3 mm**	**NED, 12 months**

NED = No Evidence of Disease.

Typically presenting as a slow-growing, asymptomatic nodule, CS may go unnoticed for years, as in our patient. Most lesions are small (<2 cm) and superficial, limiting imaging needs [2]. In our case, the lesion measured 1.6 cm and was subcutaneous, justifying direct surgical management without imaging.

Histologically, CS is characterized by its biphasic composition of epithelial and mesenchymal elements. This feature can mimic pleomorphic adenoma, especially in the upper lip, where minor salivary glands are present. Immunohistochemistry (S-100 and AE1/AE3) confirms biphasic differentiation [8, 9].

Malignant transformation of CS is extremely rare but must be considered. Malignant chondroid syringoma (MCS) typically presents with larger lesions (>3 cm), infiltrative margins, cytologic atypia, necrosis, or increased mitotic activity [10]. None of these features were observed in our case.

Surgical excision with adequate margins remains the standard treatment. While there are no universally established margins for CS, excision with 2–5 mm margins is commonly reported [4]. In our case, a 3 mm margin was sufficient to ensure complete removal without recurrence at 1 year. The use of Vicryl Rapide sutures contributed to an excellent aesthetic and functional result by minimizing suture marks and preserving upper lip mobility and symmetry—critical considerations in facial surgery [3].

Follow-up remains essential due to the risk, albeit low, of late recurrence. Most reported recurrences occurred beyond 12 months, suggesting surveillance for at least 2–5 years is advisable [11, 12].

## Conclusion

Chondroid syringoma of the upper lip is extremely rare and can mimic more common cutaneous or salivary gland tumors. Accurate diagnosis relies on histopathology supported by immunohistochemistry. Complete surgical excision with appropriate margins offers an excellent prognosis. The use of rapid-absorbing sutures like Vicryl Rapide optimizes aesthetic outcomes in delicate facial regions. Meticulous surgery ensures favorable functional/aesthetic outcomes. Long-term follow-up is essential to detect any delayed recurrence.
